# Capture of intraocular lens optic by residual capsular opening in secondary implantation: long-term follow-up

**DOI:** 10.1186/s12886-018-0741-2

**Published:** 2018-04-02

**Authors:** Tian Tian, Chunli Chen, Haiying Jin, Lyu Jiao, Qi Zhang, Peiquan Zhao

**Affiliations:** 1 Department of Ophthalmology, Xinhua Hospital, Affiliated to Medicine School of Shanghai Jiaotong University, No. 1665, Kongjiang Road, Shanghai, 200092 China; 2 Department of Ophthalmology, Shengli Oilfield Central Hospital, No.31, Jinan Road, Dong Ying, Shandong China

**Keywords:** Intraocular lens, Optic capture, Dislocation, Secondary IOL implantation

## Abstract

**Background:**

To introduce a novel surgical technique for optic capture by residual capsular opening in secondary intraocular lens (IOL) implantation and to report the outcomes of a long follow-up.

**Methods:**

Twenty patients (20 eyes) who had received secondary IOL implantation with the optic capture technique were retrospectively reviewed. We used the residual capsular opening for capturing the optic and inserted the haptics in the sulcus during surgery. Baseline clinical characteristics and surgical outcomes, including best-corrected visual acuity (BCVA), refractive status, and IOL position were recorded. The postoperative location and stability of IOL were evaluated using the ultrasound biomicroscopy.

**Results:**

Optic capture technique was successfully performed in all cases, including 5 cases with large area of posterior capsular opacity, 6 cases with posterior capsular tear or rupture,and 9 cases with adhesive capsules. BCVA improved from 0.60 logMAR at baseline to 0.36 logMAR at the last follow-up (*P* < 0.001). Spherical equivalent changed from 10.67 ± 4.59 D at baseline to 0.12 ± 1.35 D at 6 months postoperatively (*P* < 0.001). Centered IOLs were observed in all cases and remained captured through residual capsular opening in 19 (95%) eyes at the last follow-up. In one case, the captured optic of IOL slid into ciliary sulcus at 7 months postoperatively. No other postoperative complications were observed in any cases.

**Conclusions:**

This optic capture technique by using residual capsule opening is an efficacious and safe technique and can achieve IOL stability in the long follow-up.

## Background

Posterior chamber intraocular lens (IOL) are mostly implanted in the capsular bag. However, the status of lens capsules may not be sufficient to support IOL intracapsular bag implantation, especially during secondary procedure. Under these challenging and complicated situations, variously substituted IOL implanting techniques have been reported, including sulcus-IOL implantation, using an anterior chamber IOL, an iris-fixed IOL, and a transscleral-fixed posterior chamber IOL. However, each of these techniques has postoperative problems and complications. The major complications are postoperative IOL instability, dislocation, tilting, and pupillary capture of IOL [1–5].

Compared to these substituted IOL implanting techniques mentioned above, our optic capture technique was a simple and safe choice in cases with intact residual capsular opening during the secondary IOL implantation. The concept of our optic capture technique was derived from “the rhexis-fixed Lens” which was firstly described by Neuhann for placing the haptics in the sulcus and then capturing the IOL optic through the anterior continuous curvilinear capsulorhexis (CCC) opening [6]. The concept of optic capture technique was firstly described by Gimbel and DeBroff to maintain a clear visual axis in pediatric IOL surgery with the haptics in the capsular bag and optic through a posterior curvilinear capsulorhexis opening [7]. Even the optic capture technique of IOL has been described, however, the optic capture technique are mainly used in primary pediatric or adult cataract surgery. As known, the capsule remained is different one from that at primary surgery, such as fibrosis, adherent anterior and posterior capsule, and membrane-like formation. Will the residual capsular membrane offer enough strength to capture the optic of IOL? Will the IOL achieved stability in the cases with intact residual capsular opening in the long-term? However, there existed few clinical studies to manifest the safety of optic capture technique during the secondary IOL implantation. In order to answer those questions we did the present clinical study.

## Methods

### Patients

**Fig. 1:**
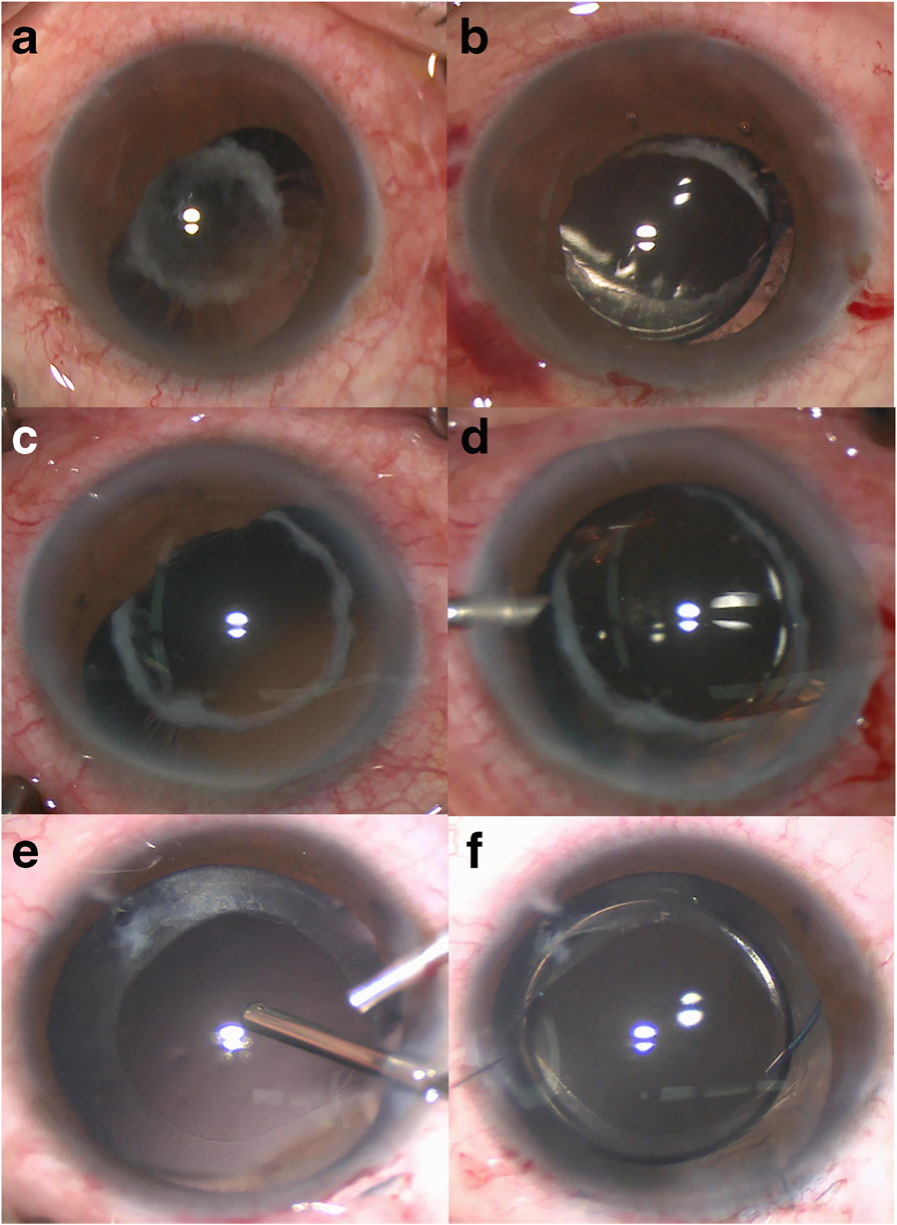
Three cases underwent optic capture technique with three indications: intraoperative photos. The intraoperative photo (**a**) showed the large area of posterior capsular opacity in a 68 years old female (Case 1) with proliferative diabetic retinopathy. Optic was captured through the residual capsular opening after posterior capsule cut using vitreous cutter (**b**). The intraoperative photo (**c**) showed 360-degree synechia of capsules and posterior synechia of iris in a 61 years old male (Case 4). After managing the posterior synechia, the optic was captured through the residual capsular opening (**d**). The intraoperative photo (**e**) showed the posterior capsule tear caused by trauma in a 12-year old female (Case 2). After trimming, the posterior capsular opening was equal to the anterior capsular opening (**e**). The captured optic was centered with clear visual axis (**f**)

This study adhered to the tenets of the Declaration of Helsinki and was approved by institution review board of Xinhua hospital affiliated to medical college, Shanghai Jiaotong University. We retrospectively reviewed 20 patients (20 eyes) who had received secondary IOL implantation with optic capture technique from April 2012 to January 2016. Surgeries were performed by one surgeon (P.Q.Z). Inclusion criteria were 1) Large area of posterior capsular opacity (PCO); severe synechia of anterior and posterior capsules; posterior capsular tear or rupture with inadequate support of capsular bag (Fig. 1). The residual capsular opening should be intact and the size should be 4.0 mm to 5.0 mm approximately. Exclusion criteria were 1) IOL can be implanted in capsular bags. 2)No enough residual capsules, which made the IOL optic capture impossible. 3) Eyes with lax zonules or zonular dehiscence. 4) Eyes with anterior megalophthalmos. 5)Axial length is longer than 28 mm.

All 20 patients had a complete ophthalmologic examination including best-corrected visual acuity (BCVA), refractive status, axial length, B-scan, intraocular pressure (IOP), endothelial cell count,slit-lamp examination, Refractive status, and dilated fundus examination pre- and postoperatively. We collected refractive status for statistical analysis at 6 months after surgery when the position of IOL and the status of anterior segment tend to be stable. BCVA was measured among 18 patients with Snellen chart and converted to logarithm of the minimum angle of resolution values (logMAR) for the statistical analysis. The remaining two patients were too young to cooperate with BCVA examination. Refractive status was measured through retinoscopy with instillation of a combination of tropicamide 1%, phenylephrine 2.5% and cyclopentolate 1%. Chloral hydrate was used in pediatric patients who were uncooperative. B scans were obtained in all 20 patients (Digital B 2000 and Ultrascan Imaging System; Alcon). Axial length was measured by optical biometer (Ver 5.4) (Carl Zeiss Meditec AG, Jena, Germany) or A scan (Digital B 2000 and Ultrascan Imaging System; Alcon). The capsules status was recorded according to intraoperative videos. The postoperative location and stability of IOL were evaluated by using the ultrasound biomicroscopy (UBM, Paradigm Medical Industries, Salt Lake City, UT). Corneal endothelial cell count was assessed with the EM-3000 (TOMEY, Nagoya, Japan) in patients who were older than 10 years pre- and postoperatively (6 months).

### Surgical technique

**Fig. 2:**
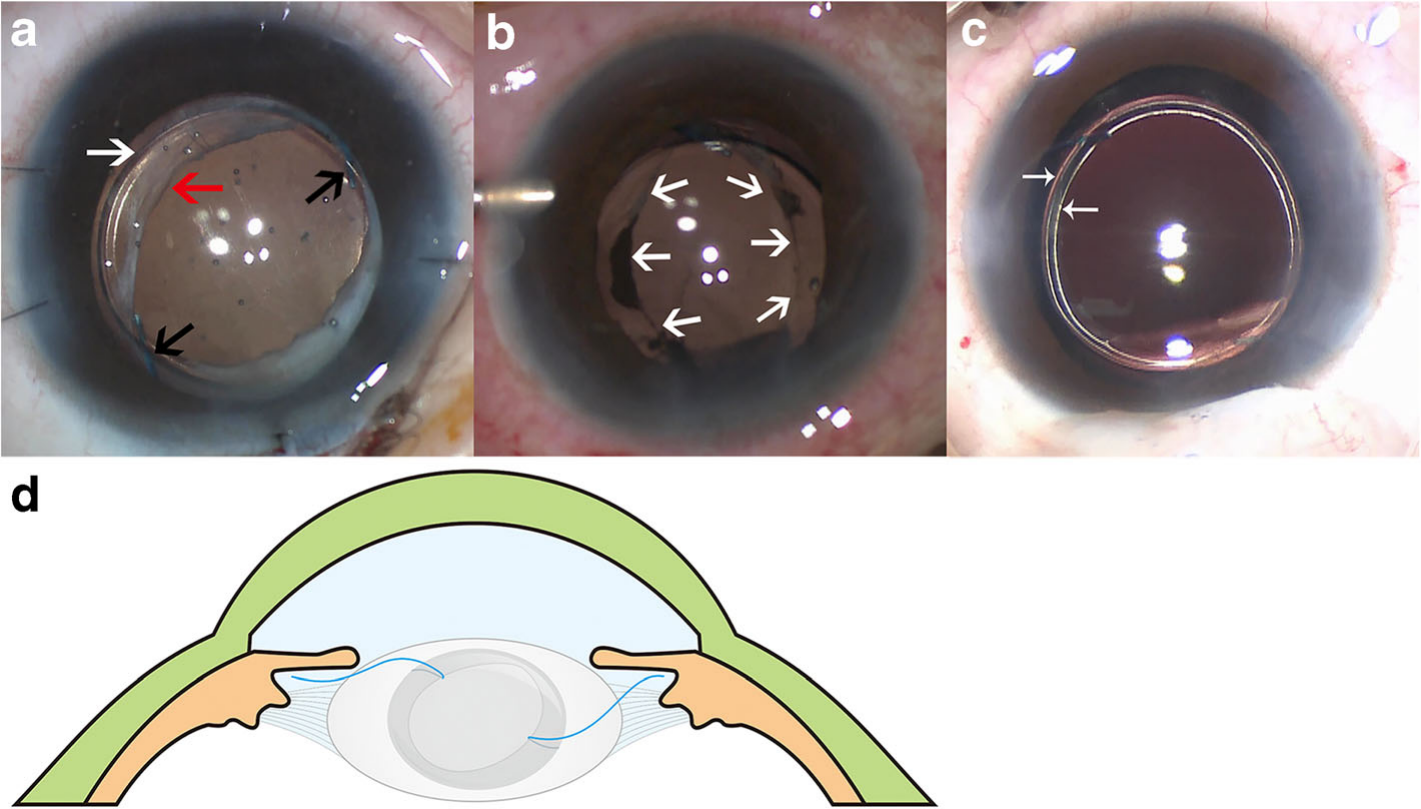
Three patients who underwent IOL optic capture technique: intraoperative photos (**a**, **b** and **c**) and schematic illustration (**d**). The intraoperative photo (**a**) showed two haptics (black arrows) of IOL were inserted in the ciliary sulcus with the optic (white arrow) captured through residual capsular openings (red arrow). And the intraoperative photo (**b**) showed the successful captured optic made an oval capsular configuration (white arrows). The ideal size of capsular opening is around 4.0 mm to 5.0 mm, which should be at least 1.0 mm or 2.0 mm (white arrows) smaller than the optic diameter (**c**). The Schematic illustrations (**d**) of optic capture technique showed the optic of IOL (the edge was shown as dark gray color) captured through residual capsular opening with haptics in the ciliary sulcus

After retrobulbar or general anesthesia was attained, an infusion cannula connected to a balanced salt plus solution (Alcon, Laboratories, Inc) was inserted into anterior chamber through infratemporal corneal incision. In cases with posterior capsule tear caused by trauma or inadvertent rupture in primary surgeries, the tear or rupture was converted to a circular, well-centered capsular opening as much as possible. In cases with large area of PCO, posterior capsulorhexis was performed with virtrectomy cutter. Posterior synechia of iris was meticulously dissected with assistance of viscoelastic if needed. After managing capsules properly, a foldable IOL with a 6.0 mm optic and 13.0 mm haptic diameter (Tecnis ZA9003; AMO, Santa Ana, CA) was inserted into the anterior chamber through a 2.8 mm superior clear corneal incision. Residual capsular opening should be large enough to allow the IOL optic to pass through and small enough to capture the optic. The appropriate size of residual capsular opening was 4.0–5.0 mm approximately. Then, one haptic of the IOL was inserted into the ciliary sulcus with a Sinskey hook, and then the other haptic was positioned in contralateral ciliary sulcus in the same manner. The positions of haptics should avoid the capsular defect area. After confirming the positions of the two haptics, one side of the optic was then captured through residual capsular opening, and the other side was pressed in the same manner. The successfully captured optic made an oval capsular configuration (Fig. 2). Finally, the corneoscleral incisions were closed with a single 10–0 nylonsuture if necessary.

### Statistical analysis

Statistical analyses were performed using SPSS version 19 for Windows (SPSS Inc., Chicago, USA). Paired *t*-test was conducted in this study. *P* value less than 0.05 (two tails) was considered as statistical difference.

## Results

Successful optic capture IOL implantation was achieved in all 20 aphakic eyes (20 patients). The mean age was 31.95 ± 26.83 years. The capsular status was collected based on the surgery video, including 5 eyes (25%) with large area of posterior capsular opacity, 6 eyes (30%) with posterior capsular tear or rupture,and 9 eyes (45%) with adhesive capsules. Fifteen eyes (75%) combined with retinal disorders. The mean follow-up was 24.51 ± 13.47 months. The details and clinical characteristics of the 20 patients were shown in Table 1.

**Table 1:** Details and characteristics of patients who underwent secondary IOL implantation with optic capture

PT	Age (Y) / Sex	Eye	Preoperative diagnosis	History of previous operation(s)	Capsular status	BCVA pre/post	Follow-up (months)
1	68/F	OD	PDR	Phaco+PPV + C3F8	Large PCO	0.1/0.3	18
2	12/F	OD	Traumatic cataract	Lensectomy	PC tear	0.05/0.15	9
3	5/F	OD	Congenital cataract	Lensectomy+ anterior PPV	Adhesive	FC/0.1	7
4	61/M	OS	RRD	Phaco+PPV + C3F8	Adhesive	FC/0.05	26
5	1/F	OD	PHPV	Lensectomy	Large PCO	Uncooperated/ Uncooperated	6
6	35/M	OD	RRD	Lensectomy+PPV + C3F8	PC rupture	0.01/0.1	27
7	4/M	OD	Traumatic cataract	Lensectomy	Adhesive	Uncooperated/0.12	16
8	4/F	OD	Congenital cataract	Lensectomy	Adhesive	HM/FC	13
9	61/M	OS	PDR	Phaco+PPV + C3F8	Large PCO	FC/0.1	32
10	4/F	OD	PHPV; Concurrent cataract	Lensectomy	Adhesive	HM/0.12	15
11	2/M	OD	PHPV; Concurrent cataract	Lensectomy	Adhesive	Uncooperated/ Uncooperated	8
12	46/M	OD	PDR	Phaco+PPV + C3F8	Adhesive	FC/0.3	41
13	81/M	OS	Age related cataract	Phaco	PC rupture	0.08/0.20	12
14	62/F	OD	ERM; Concurrent cataract	Phaco+PPV + ILM peeling+C3F8	Large PCO	0.1/0.3	28
15	40/F	OS	RRD	Lensectomy+PPV + C3F8	PC rupture	0.3/0.8	35
16	2/F	OS	PHPV; Concurrent cataract	Lensectomy+ anterior PPV	Adhesive	Uncooperated/ Uncooperated	6
17	10/M	OS	Traumatic macular hole	Lensectomy + PPV	Adhesive	FC/0.1	18
18	53/F	OD	RRD	Phaco +PPV+ C3F8	PC rupture	FC/0.6	32
19	58/M	OS	ERM; Concurrent cataract	Phaco+PPV + C3F8	Large PCO	0.03/0.5	46
20	50/M	OS	Macular hole; Concurrent cataract	Phaco+PPV + ILM peeling+C3F8	PC rupture	FC/0.25	35

*PT* patient, *M* male, *F* female, *PDR* proliferative diabetic retinopathy, *RRD* rhegmatogenous retinal detachment, *PHPV* persistent hyperplasia of primary vitreous, *ERM* epiretinal retinal membrane, *PPV* pars plana vitrectomy, *PRP* panretinal photocoagulation, *ILM* internal limiting membrane, *PCO* posterior capsular opacity, *PC* posterior capsule, *BCVA* best corrected visual acuity, *Pre* preoperation, *Post* postoperation, *HM* hand motion, *FC* figure counting, *IOL* intraocular lens

**Fig. 3:**
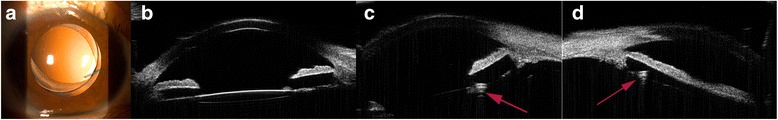
A 50 years old male (Case 20), phaco and vitrectomy were performed because of macular hole. The Slit-lap photo (**a**) showed the centered IOL with optic captured through posterior capsular opening and haptics in the sulcus, at 6 months postoperatively. Ultrasound biomircoscopy (**b**, **c** and **d**) showed the optic was centered and two haptics were located at 2 o’clock and 8 o’clock, repectively

**Fig. 4:**
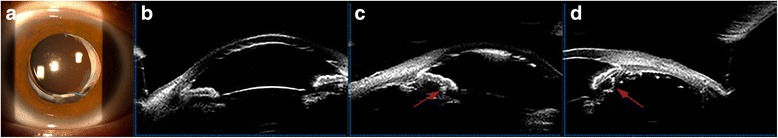
A 10 years old male (Case 17), lensectomy and pans plana vitrectomy were performed because of traumatic macular hole. The Slit-lap photo (**a**) showed the centered IOL at 7 months postoperatively. However, ultrasound biomircoscopy (**b**, **c** and **d**) showed the optic and two haptics were in the sulcus. Two haptics were located at 5 o’clock and 11 o’clock, respectively

BCVA improved from 0.60 logMAR at baseline to 0.36 logMAR at the last follow-up (*P* < 0.001). Spherical equivalent changed from 10.67 ± 4.59 D at baseline to 0.12 ± 1.35 D at 6 months postoperatively. The position of IOL remained captured through residual capsular opening in 19 (95%) eyes at the last follow-up (Fig. 3). In one case, the captured optic of IOL slid into ciliary sulcus at 7 months postoperatively (Fig. 4). The surgical outcomes were shown in Table 2. No patients had complaints of dazzle or other visual disorders. Iris synechia, anterior cells, anterior uveitis and secondary glaucoma were not observed in any cases. No other related complications were found in any case at the last follow-up.

**Table 2:** Surgical outcomes of secondary IOL implantation with optic capture

Parameter	Mean ± SD	*P* value
BCVA, log MAR		< 0.001
Preoperative	0.60 ± 0.44	
Postoperative	0.36 ± 0.17	
Spherical equivalent, (D)		< 0.001
Preoperative	10.67 ± 4.59	
Postoperative	0.12 ± 1.35	
IOL position, n (%)		
Captured	19 (95%)	
Ciliary sulcus	1 (5%)	
Endothelial Cell Count		0.431
Preoperative	2326 ± 423	
Postoperative	2158 ± 389	
IOP		0.524
Preoperative	15.26 ± 3.65	
Postoperative	14.86 ± 2.82	

*BCVA* best corrected visual acuity, *IOL* intraocular lens, *IOP* intraocular ocular pressure

## Discussion

The present study described the application of optic capture technique in eyes with intact residual capsular opening during the secondary implantation. Our study demonstrated the safety and efficiency of optic capture technique; good long-term visual outcome; clinically centered IOL, and no secondary opacification of the visual axis at the mean follow-up of 23.51 months. However, the optic of IOL may slide into sulcus during the follow-up.

The situation of lens capsules may be complicated and challenging in the secondary IOL implantations, such as no adequate support of capsular bag, large area of posterior capsular opacity (PCO) and serious synechia of anterior and posterior capsules that lead no potential space for the in-the-bag IOL implantation. Until now, there is no consensus on the optimal choice of IOL implantation methods in eyes within these complicated situations of lens capsules mentioned above. In eyes with intact anterior CCC, the sulcus-fixation is always substitute for capsular bag. However, it has been reported that the implantation of foldable IOLs into the ciliary sulcus may be related to a higher rate of decentration [5]. Besides, the incidence of pupillary capture of the sulcus-fixation IOL is raised when combined with pars planar vitrectomy and gas tamponade [8]. Moreover, the surgeon personally encountered frequent and recurrent pupillary capture of the sulcus-fixation IOL optic after phacovitrectomy. All these results demonstrated the instability of ciliary sulcus inserted IOL in eyes with posterior capsule rupture or larger PCO. Other surgeons may consider transscleral-fixed IOL, iris-fixed IOL or anterior chamber IOL in cases without adequate capsular support. Each technique has advantages and disadvantages. Several postoperative complications have been reported with these techniques, including retinal detachment, vitreous hemorrhage, endophthalmitis, IOL dislocation, and pupillary capture [4, 9]. It has been reported that pupillary capture of the IOL optics could occurred in 7.9% to 14.3% of cases after scleral-fixated sutured posterior chamber IOL (PC IOL) implantation [3, 10]. Dong Jin Kang et al. have reported that five eyes (7.8%) had pupillary capture after transscleral IOL fixation. Other complications after transscleral fixation were vitreous hemorrhage in 5 eyes (7.8%) and IOP elevation in 8 eyes (12%) [11]. Besides, suturing of the haptics to the sclera may result in suture erosion, delayed IOL dislocation owing to suture breakage, or suture exposure-induced endophthalmitis [4, 12].

Compared to these techniques, our optic capture technique by residual capsular opening offers a tight seal of the IOL-capsule diaphragm, it helps maintain stable compartmentalization between the anterior and posterior segments of the eye and reduces the rate of postoperative IOL dislocation significantly. Besides, the optic capture procedure was simple with short learning curve. The variations of optic capture include (a) haptics in the sulcus and IOL optic capture through a CCC, (b) haptics in the sulcus and IOL optic capture through an anterior capsule opening and a posterior CCC (PCCC), (c) haptics in the capsular bag and IOL optic capture through a PCCC, (d) haptics in the capsular bag and IOL optic capture through an anterior CCC, (e) haptics in the sulcus and IOL capture through a capsular membrane opening, and (f) haptics posterior to the capsular bag and IOL capture through a capsular membrane opening [7]. In our study, the surgeon placed the haptics in the sulcus and optic captured through the residual capsular opening. To obtain successful optic capture in the secondary IOL implantation, trimming the residual capsular membranes to fit to capture the optic of IOL was the key factor. The ideal capsular opening is around 4.0 mm to 5.0 mm in primary surgery, which should be at least 1.0 mm or 2.0 mm smaller than the optic diameter but not too small [13]. In this study, we found that even the posterior capsular opening or tear was not entirely concentric, the captured optic still could achieve centered in the visual axis. It may because as long as the position of two haptics were symmetrical,the haptic-optic junction could offer a tight seal that maintain the optic centered in the visual axis. In our study, clinically centered IOLs were observed in all cases, and the optic edge was not seen through an undilated pupil at the last follow-up. In one case, however, the captured optic of IOL slid into ciliary sulcus at 7 months postoperatively. The UBM and dilated slit-lamp examination showed that the haptics and optic of IOL were in ciliary sulcus. In this case, the residual capsular opening was just approximately 1.0 mm smaller than optic diameter and completely round-shape. We hypothesized the capsular membrane became stiff after primary surgery and the size of the capsular opening was not small enough so that did not off a tight enough haptic-optic junction. Thus, we suggested the residual capsular opening should be less than 5.0 mm in cases with stiff capsular membrane during secondary IOL implantation. As the optic was still centered in the visual axis and BCVA was not impaired, the second operation was not performed to this patient.

The other advantage of optic capture technique is avoiding secondary opacification of the visual axis that may be caused by proliferation of Elschnig pearls. In our study, the surgeon placed the haptics in the sulcus and optic captured through the posterior opening, leading to apposition of anterior and posterior capsule leaflets anterior to the IOL optic. Compared to place two haptics in the capsular bag, the sulcus-placed haptics, which made a 360-degree seal of apposed capsule leaflets, avoided lens epithelial cells transdifferentiation and Elsching pearls releasing. Consequently, the rate of capsular shrinkage and visual axis opacification will be decreased significantly. In our study, capsular shrinkage and visual axis opacification were not observed in any case at the last follow-up. The BCVA improved from preoperative 0.60 ± 0.44 to postoperative 0.36 ± 0.17 logMAR. Because of primary ocular diseases, the BCVA may have a limited improvement in some cases.

The optic capture technique has limitations. It should not be performed if posterior capsular opening was quite eccentric or the size was not fit to capture the optic. Besides, the technique may not be ideal for eyes with anterior megalophthalmos. The larger capsular bags in these cases may increase the rate of IOL decentration.

## Conclusions

In summary, optic capture through the residual capsular opening may be an efficacious and safe technique to achieve IOL stability in eyes with challenging capsular status during secondary IOL implantation. Attention should be paid to the cases with stiff capsular membrane when performed optic capture. Additional study with more cases and further follow-up is needed to manifest the safety and long-term efficacy before recommending the widespread application of this technique during secondary IOL implantation with challenging capsular status.

## Abbreviations

BCVA: Best-corrected visual acuity
CCC: Continuous curvilinear capsulorhexis
IOL: Intraocular lens
IOP: Intraocular pressure
logMAR: Logarithm of the minimum angle of resolution values
PCO: Posterior capsular opacity
UBM: Ultrasound biomicroscopy
